# Association between dietary intake and risk of Parkinson’s disease: cross-sectional analysis of survey data from NHANES 2007–2016

**DOI:** 10.3389/fnut.2023.1278128

**Published:** 2023-12-15

**Authors:** Ling Liu, Qiuyan Shen, Yi Bao, Fang Xu, Dan Zhang, Hongyan Huang, Liangdan Tu, Yanming Xu

**Affiliations:** Department of Neurology, West China Hospital, Sichuan University, Chengdu, Sichuan, China

**Keywords:** diet, Parkinson’s disease, NHANES, micronutrients, dietary intake

## Abstract

**Background:**

While dietary factors have shown an association with Parkinson’s disease (PD), the available data remains a subject of ongoing debate and controversy.

**Aim:**

We sought to evaluate potential relationships between dietary consumption of nutrients and micronutrients and risk of PD in a large sample.

**Methods:**

Cross-sectional data were retrospectively analyzed for 10,651 adults aged 40–80 years that had been collected in the US between 2007 and 2016 as a component of the nationwide National Health and Nutrition Examination Survey. Aspects of dietary intake were compared between those who reported having specific PD medication regimens or not when they completed the survey, and potential associations between diet and risk of PD were explored using binomial logistic regression. We employed Propensity Score Matching (PSM) to minimize the impact of potential confounding factors, thus enhancing the reliability of the results. Additionally, subgroup analysis based on gender and age was conducted to investigate these relationships.

**Results:**

Higher dietary intake of iron was linked to greater PD risk [odds ratio (OR) 1.065, 95% confidence interval (CI) 1.019–1.114, *p* = 0.006], whereas risk decreased with higher intake of vitamin K (OR 0.999, 95% CI 0.998–1.000, *p* = 0.024) or vitamin C (OR 0.998, 95% CI 0.996–0.999, *p* = 0.039). Even after applying PSM, the connection between dietary iron intake and dietary vitamin C intake with PD risk remained substantial. Subgroup analysis results revealed a significant positive association between dietary intake of iron from food and the PD risk, which was evident among individuals under 60 years of age and among males.

**Conclusion:**

The intake of micronutrients can influence risk of PD, which should be verified and explored further in prospective samples with other dietary habits and ethnic backgrounds.

## 1 Introduction

Parkinson’s disease (PD) is one of the most common neurodegenerative disorders (1), affecting roughly 1.5% of the general population older than 65 years and 3.0% of the population older than 80 years (2). Incidence is expected to rise as the global population ages (3). In PD, selective degeneration of dopaminergic cells in the substantia nigra of the brain cause motor symptoms such as bradykinesia, postural instability, resting tremor, and rigidity, as well as non-motor symptoms such as hyposmia, sleep disorders, cognitive impairment, and autonomic dysfunction. Both types of symptoms can substantially reduce one’s quality of life and ability to live independently.

The etiology of Parkinson’s disease (PD) is widely acknowledged to be multifactorial, encompassing a combination of genetic and environmental factors (4, 5). Among these environmental factors, diet has emerged as a significant determinant. Research indicates that adopting a diet rich in antioxidants (6), or adhering to a “Mediterranean” dietary pattern characterized by an abundance of plant-based foods, fish, and olive oil (7), may potentially mitigate the risk of PD. In contrast, a diet high in cholesterol and saturated fat is associated with an increased risk of PD (8). These factors were also found to potentially influence the clinical features of PD (9). Most of this work has been conducted on relatively small or medium-sized samples, which may limit its generalizability. Moreover, in these studies, the findings regarding the connections between dietary consumption and PD were inconclusive.

To complement this evidence base, we investigated potential correlations between dietary elements and the risk of PD within a substantial sample extracted from the U.S. National Health and Nutrition Examination Survey (NHANES) (10). The present work appears to be the first use of NHANES data for exploring links between diet and PD.

## 2 Methods

### 2.1 Sample and survey

**Figure 1:**
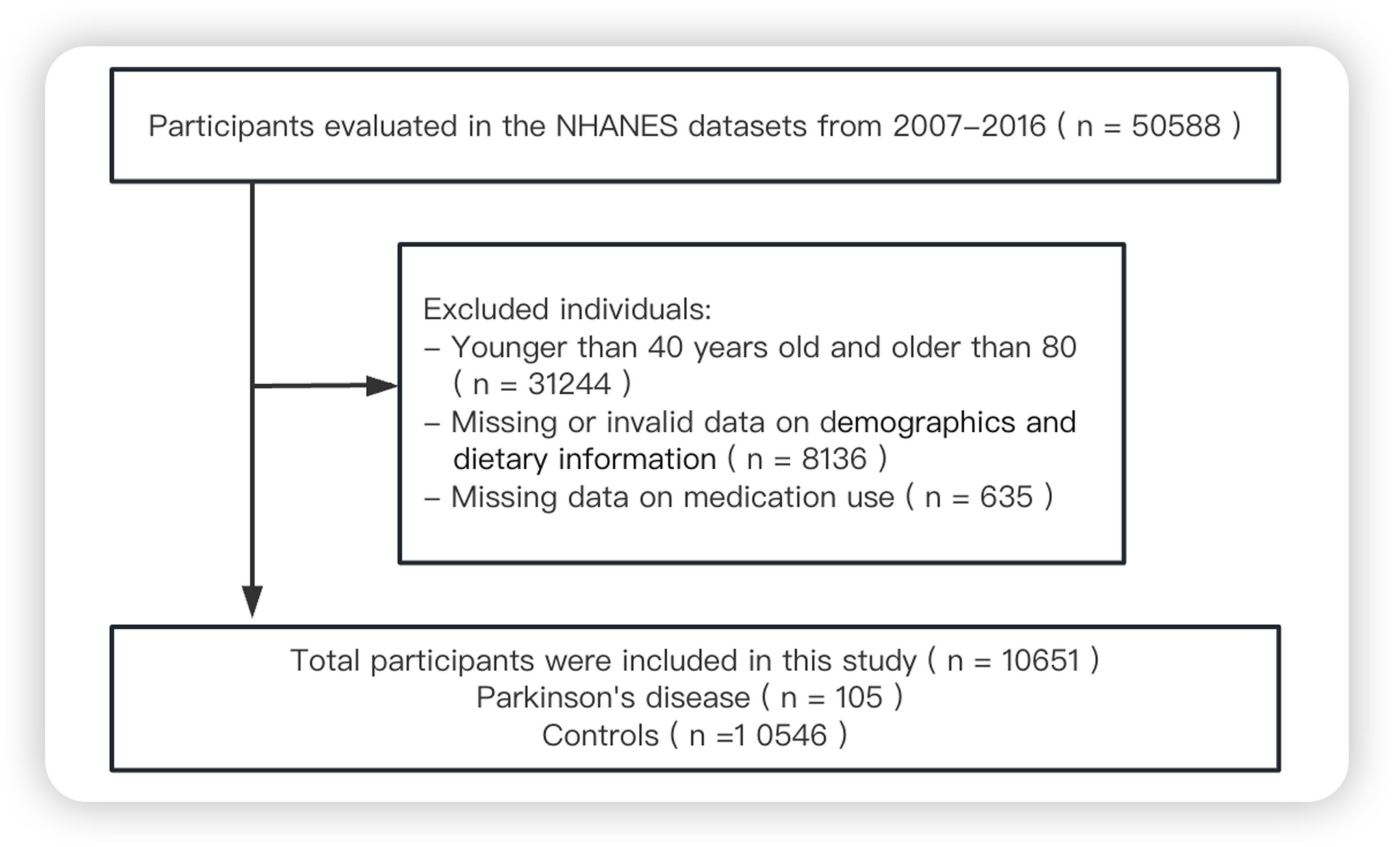
Flowchart of study participants.

Since 1999, data on the demographics, socioeconomic status, health and diet of adults and children in the US general population have been collected through the NHANES, conducted by the National Center for Health Statistics at the Centers for Disease Control and Prevention (11). For the present study, we screened data from 50,588 respondents who participated in the NHANES in 2007–2008, 2009–2010, 2011–2012, 2013–2014 or 2015–2016. We excluded individuals younger than 40 years or older than 80 years, those with missing survey data, and those who reported taking “unsecure” PD medications (cabergoline, orphenadrine or pramipexole) (12), leaving 10,651 in the final analysis (Figure 1).

### 2.2 Definition of PD

Survey respondents were considered to have PD if they reported taking appropriate medications, defined as carbidopa, levodopa, methyldopa, benztropine, ropinirole, entacapone, and amantadine (12, 13).

### 2.3 Dietary intakes

Dietary information of respondents in each cycle was collected through 24-h diet recall interviews conducted in-person in a “mobile examination center,” followed 3–10 days later by a second diet recall interview conducted by telephone. Dietary intakes were determined by averaging data from two dietary recalls, when such information was accessible; otherwise, data from a single dietary recall were utilized. The 24-h dietary supplement usage component was administered subsequent to the 24-h dietary interview pertaining to food and beverage consumption. In the case of dietary supplements, the total intake was also computed as an average of data from two dietary recalls, when available. We further estimated total nutrient intakes by summing the nutrient intakes from food sources and dietary supplements. The survey questionnaires can be found in the supplementary materials.

### 2.4 Demographics, income, lifestyle, and comorbidities

NHANES gathered demographic information, including age, gender, ethnic background, educational level, poverty-income ratio, smoking habits, and alcohol consumption, through household interviews. In addition, data related to body weight and height were obtained during physical examinations conducted at the mobile examination center. Participants were categorized according to their education level, which included “below high school,” “high school,” or “above high school.” Body mass index was computed as the quotient of an individual’s weight in kilograms divided by the square of their height in meters. Respondents were categorized as “former smokers” if they reported a history of a minimum of 100 cigarettes in the past but were not currently smoking, and as “current smokers” if they reported a history of at least 100 cigarettes in the past and were presently smoking. In terms of alcohol consumption, participants were categorized as “former drinkers” if they reported having consumed a minimum of 12 alcoholic drinks per year in the past but fewer than 12 in the preceding year, and as “current drinkers” if they reported consuming at least 12 alcoholic drinks per year in the past, including the most recent year. Respondents were categorized by their coffee intake into two groups: < 100 mg/d or ≥ 100 mg/d. Respondents were considered to have comorbidities if they reported having been diagnosed with hypertension or diabetes.

### 2.5 Statistical analysis

Continuous data were presented as mean ± standard deviation, and categorical data were reported as n (%). Statistical significance of differences in survey variables between respondents with and without PD was assessed using appropriate statistical tests, including Student’s t-test, the Mann–Whitney U rank sum test, or the χ2 test. Factors potentially associated with PD risk were explored using binomial logistic regression. In all analyses, we applied study-specific dietary sample weights to accommodate the intricate sample design of NHANES (14). To mitigate differences between PD patients and non-PD participants, a 1:4 ratio PSM analysis was employed. This process entailed controlling for multiple confounding variables, including as age, sex, body mass index, poverty-income ratio, education, ethnicity, smoking habits, alcohol intake, hypertension, diabetes, and caffeine intake. In addition, we also performed subgroup analysis based on gender and age to investigate the relationship between dietary intakes and PD risk. Data were analyzed using R version 4.1.2 and Stata version 17.0. Results, when applicable, were reported as odds ratios (ORs) along with their corresponding 95% confidence intervals (CIs).

## 3 Results

### 3.1 Characteristics of included participants

Out of the 10,651 participants, 105 (0.986%) were found to have PD. Compared to respondents without PD, those with PD had a considerably lower level of education (*p* = 0.003) and lower alcohol intake (*p* = 0.035), but significantly higher rates of hypertension (*p* = 0.002) and diabetes (*p* = 0.030) (Table 1).

**Table 1:** Study population, stratified by Parkinson’s disease (PD) status.

Variable	Total	No PD	PD	*p*
	(*N* = 10,651)	(*n* = 10,546)	(*n* = 105)	
Age, yr	57.235 ± 11.327	57.258 ± 11.328	55.367 ± 11.084	0.056
Sex				0.597
Male	49.438	49.409	51.719	
Female	50.562	50.591	48.281	
Body mass index	29.681 ± 6.653	29.675 ± 6.654	30.106 ± 6.582	0.459
Poverty-income ratio	2.735 ± 1.638	2.732 ± 1.636	2.919 ± 1.733	0.192
Education level				0.003
Below high school	14.264	14.225	17.31	
High school	22.503	22.658	10.193	
Above high school	63.233	63.117	72.497	
Ethnicity				0.266
Mexican American	5.418	5.437	3.911	
Other Hispanic	4.022	4.033	3.132	
Non-Hispanic White	75.914	75.821	83.248	
Non-Hispanic Black	9.33	9.392	4.447	
Other	5.316	5.317	5.263	
Smoking				0.699
Non-smoker	47.509	47.507	47.703	
Former smoker	33.72	33.753	31.063	
Current smoker	18.771	18.74	21.233	
Alcohol intake				0.035
Non-drinker	19.365	19.365	19.38	
Former drinker	77.466	77.515	73.549	
Current drinker	3.169	3.120	7.071	
Hypertension				0.002
Yes	42.518	42.683	29.444	
No	57.482	57.317	70.556	
Diabetes				0.030
Yes	13.016	13.095	6.721	
No	86.984	86.905	93.279	
Caffeine intake, mg/d				0.059
≥ 100	64.512	72.298	64.414	

Values are % or mean ± standard error, unless otherwise noted.

### 3.2 Dietary intakes

Table 2 presents the dietary intakes from foods of the included participants before PSM. Patients suffering from PD took less lycopene (*p* = 0.034) and more vitamin B1 (*p* = 0.016), vitamin B2 (*p* = 0.014), niacin (*p* = 0.020), vitamin B6 (*p* = 0.004), folic acid (*p* = 0.049) and iron (*p* < 0.001). Participants in the no-PD group demonstrated a notably higher propensity for the utilization of dietary supplements (*p* = 0.001). And PD patients tend to take less vitamin C than controls (*p* = 0.021) (Supplementary Table S1). Additionally, the total intake of lycopene (*p* = 0.032) and vitamin C (*p* = 0.021) among PD patients is lower than controls. There was no statistically significant difference observed in the other dietary factors with PD (*p* > 0.05) (see Table 3).

**Table 2:** Dietary intakes from food by study participants, stratified by Parkinson’s disease (PD) status.

Variable	Total	No PD	PD	*p*
	(*N* = 10,651)	(*n* = 10,546)	(*n* = 105)	
Energy, kcal/d	2055.592 ± 760.334	2054.765 ± 755.695	2121.267 ± 1063.822	0.317
Protein, g/d	81.060 ± 32.225	81.068 ± 32.217	80.443 ± 32.900	0.824
Sugar, g/d	106.034 ± 60.189	105.972 ± 59.823	110.925 ± 84.208	0.346
Fiber, g/d	17.422 ± 9.006	17.409 ± 9.009	18.468 ± 8.727	0.178
Fat, g/d	80.241 ± 36.684	80.239 ± 36.673	80.365 ± 37.481	0.969
Cholesterol, mg/d	288.133 ± 178.472	288.502 ± 178.874	258.852 ± 139.916	0.057
Retinol, μg/d	443.512 ± 463.785	442.619 ± 463.720	514.426 ± 463.399	0.076
Vitamin A, μg/d	672.890 ± 622.297	671.075 ± 621.831	816.937 ± 642.053	0.007
Beta-carotene, μg/d	2487.106 ± 4425.726	2477.691 ± 4435.025	3234.165 ± 3533.188	0.051
Beta-cryptoxanthin, μg/d	86.300 ± 171.380	86.237 ± 171.561	91.322 ± 156.339	0.734
Lycopene, μg/d	4938.227 ± 7268.409	4954.946 ± 7289.854	3611.568 ± 5125.939	0.034
Vitamin B1, mg/d	1.603 ± 0.761	1.601 ± 0.755	1.761 ± 1.147	0.016
Vitamin B2, mg/d	2.184 ± 1.007	2.182 ± 1.003	2.399 ± 1.237	0.014
Niacin, mg/d	24.960 ± 11.308	24.931 ± 11.228	27.229 ± 16.258	0.020
Vitamin B6, mg/d	2.056 ± 1.095	2.053 ± 1.089	2.329 ± 1.478	0.004
Folic acid, μg/d	171.979 ± 148.010	171.662 ± 148.188	197.114 ± 130.712	0.049
Vitamin B12, μg/d	5.146 ± 5.200	5.136 ± 5.185	5.969 ± 6.241	0.067
Vitamin C, mg/d	82.441 ± 75.471	82.463 ± 75.652	80.680 ± 59.307	0.787
Vitamin D, μg/d	4.828 ± 4.585	4.826 ± 4.587	4.986 ± 4.430	0.690
Vitamin E, mg/d	8.626 ± 5.265	8.620 ± 5.258	9.107 ± 5.801	0.290
Vitamin K, μg/d	122.420 ± 274.367	122.346 ± 275.827	128.313 ± 107.136	0.804
Calcium, mg/d	938.758 ± 477.001	938.417 ± 475.625	965.843 ± 575.147	0.511
Phosphorus, mg/d	1364.860 ± 540.032	1364.262 ± 538.117	1412.291 ± 673.223	0.309
Magnesium, mg/d	305.575 ± 128.697	305.509 ± 128.826	310.813 ± 117.907	0.637
Iron, mg/d	14.969 ± 7.584	14.928 ± 7.415	18.195 ± 15.777	<0.001
Zinc, mg/d	11.499 ± 6.172	11.497 ± 6.169	11.625 ± 6.429	0.812
Copper, mg/d	1.313 ± 0.855	1.313 ± 0.857	1.332 ± 0.622	0.792
Sodium, mg/d	3438.391 ± 1414.571	3437.458 ± 1412.253	3512.446 ± 1586.044	0.544
Potassium, mg/d	2766.180 ± 1071.077	2765.357 ± 1071.932	2831.510 ± 998.763	0.480
Selenium, μg/d	111.929 ± 49.962	111.935 ± 49.909	111.488 ± 53.974	0.918

**Table 3:** Total dietary intakes in participants with and without Parkinson’s disease.

Variable	Total	No PD	PD	*p*
	(*N* = 10,651)	(*n* = 10,546)	(*n* = 105)	
Lycopene, μg/d	5036.445 ± 7311.785	5053.544 ± 7333.691	3679.639 ± 5112.869	0.032
Vitamin B1, mg/d	4.737 ± 18.012	4.736 ± 18.070	4.803 ± 12.599	0.966
Vitamin B2, mg/d	4.505 ± 9.792	4.490 ± 9.765	5.683 ± 11.679	0.163
Niacin, mg/d	40.249 ± 82.623	40.241 ± 83.067	40.847 ± 31.531	0.933
Vitamin B6, mg/d	5.562 ± 15.078	5.563 ± 15.127	5.479 ± 10.519	0.949
Folic acid, μg/d	323.308 ± 476.121	322.924 ± 478.295	353.790 ± 247.115	0.458
Vitamin B12, μg/d	95.228 ± 892.866	95.820 ± 897.447	48.221 ± 379.695	0.542
Vitamin C, mg/d	184.484 ± 363.617	185.394 ± 365.551	112.261 ± 122.470	0.021
Vitamin D, μg/d	17.068 ± 47.666	17.105 ± 47.918	14.134 ± 18.682	0.476
Vitamin K, μg/d	134.956 ± 522.786	134.934 ± 525.932	136.695 ± 107.290	0.969
Calcium, mg/d	1123.994 ± 588.499	1123.641 ± 587.717	1151.934 ± 646.979	0.582
Phosphorus, mg/d	1374.771 ± 540.841	1374.177 ± 538.923	1421.944 ± 674.219	0.312
Magnesium, mg/d	334.432 ± 162.995	334.499 ± 163.314	329.150 ± 135.193	0.707
Iron, mg/d	18.157 ± 14.786	18.130 ± 14.756	20.271 ± 16.817	0.098
Zinc, mg/d	15.029 ± 10.767	15.043 ± 10.787	13.881 ± 8.890	0.217
Copper, mg/d	1.524 ± 1.015	1.523 ± 1.016	1.597 ± 0.891	0.404
Sodium, mg/d	3439.696 ± 1414.440	3438.771 ± 1412.127	3513.062 ± 1585.559	0.548
Potassium, mg/d	2774.416 ± 1070.217	2773.653 ± 1071.067	2834.973 ± 998.634	0.512
Selenium, μg/d	119.251 ± 499.602	119.307 ± 502.700	114.751 ± 56.637	0.917

### 3.3 Association between dietary intakes and PD risk

In our analysis of dietary intake solely from food sources, a negative correlation was identified between dietary intake of vitamin K and PD risk (OR 0.999, 95% CI 0.998–1.000, *p* = 0.024). Conversely, an increased intake of iron was related to an elevated risk of PD (OR 1.065, 95% CI 1.019–1.114, *p* = 0.006). These associations retained their statistical significance even following adjustments for potential confounding factors (Table 4; Supplementary Table S4). When considering the total dietary intake, including both food and dietary supplements, we identified a notable inverse correlation between vitamin C intake and the risk of PD (OR 0.998, 95% CI 0.996–0.999, *p* = 0.008; Table 5; Supplementary Figure S2).

**Table 4:** Associations between dietary intakes from food and risk of Parkinson’s disease (PD).

Variable	Unadjusted OR (95% CI)	*p*	Adjusted OR (95% CI)*	*p*
Energy, kcal/d	1.000 (0.999, 1.002)	0.582	1.000 (0.999, 1.002)	0.815
Protein, g/d	0.990 (0.957, 1.025)	0.583	0.990 (0.956, 1.024)	0.554
Sugar, g/d	0.998 (0.990, 1.005)	0.539	0.997 (0.990, 1.005)	0.519
Fiber, g/d	0.993 (0.945, 1.044)	0.785	1.000 (0.947, 1.055)	0.994
Fat, g/d	0.998 (0.983, 1.013)	0.788	1.001 (0.985, 1.017)	0.902
Cholesterol, mg/d	0.998 (0.995, 1.001)	0.222	0.998 (0.995, 1.001)	0.287
Retinol, μg/d	0.999 (0.994, 1.003)	0.575	0.999 (0.994, 1.004)	0.692
Vitamin A, μg/d	1.002 (0.997, 1.007)	0.556	1.001 (0.996, 1.006)	0.639
Beta-carotene, μg/d	1.000 (0.999, 1.000)	0.790	1.000 (1.000, 1.000)	0.903
Beta-cryptoxanthin, μg/d	1.000 (0.999, 1.001)	0.657	1.000 (1.000, 1.001)	0.438
Lycopene, μg/d	1.000 (1.000, 1.000)	0.100	1.000 (1.000, 1.000)	0.093
Vitamin B1, mg/d	0.779 (0.430, 1.411)	0.410	0.807 (0.439, 1.483)	0.489
Vitamin B2, mg/d	1.179 (0.556, 2.499)	0.668	1.043 (0.443, 2.452)	0.924
Niacin, mg/d	0.994 (0.909, 1.088)	0.899	0.997 (0.908, 1.095)	0.950
Vitamin B6, mg/d	1.145 (0.834, 1.572)	0.401	1.200 (0.855, 1.684)	0.291
Folic acid, μg/d	1.001 (1.000, 1.002)	0.107	1.001 (1.000, 1.002)	0.109
Vitamin B12, μg/d	1.007 (0.921, 1.100)	0.885	1.012 (0.907, 1.129)	0.835
Vitamin C, mg/d	0.999 (0.995, 1.003)	0.542	0.999 (0.996, 1.003)	0.760
Vitamin D, μg/d	0.975 (0.903, 1.053)	0.524	0.984 (0.915, 1.059)	0.671
Vitamin E, mg/d	1.023 (0.946, 1.107)	0.563	1.021 (0.945, 1.103)	0.600
Vitamin K, μg/d	0.999 (0.998, 1.000)	0.024	0.999 (0.998, 1.000)	0.021
Calcium, mg/d	0.999 (0.998, 1.001)	0.384	0.999 (0.998, 1.001)	0.414
Phosphorus, mg/d	1.001 (0.999, 1.003)	0.169	1.001 (0.999, 1.003)	0.174
Magnesium, mg/d	0.997 (0.992, 1.002)	0.178	0.996 (0.991, 1.001)	0.090
Iron, mg/d	1.065 (1.019, 1.114)	0.006	1.066 (1.020, 1.114)	0.005
Zinc, mg/d	0.940 (0.874, 1.012)	0.099	0.937 (0.870, 1.010)	0.087
Copper, mg/d	0.887 (0.617, 1.275)	0.516	0.847 (0.558, 1.285)	0.434
Sodium, mg/d	1.000 (1.000, 1.000)	0.885	1.000 (1.000, 1.000)	0.936
Potassium, mg/d	1.000 (0.999, 1.001)	0.724	1.000 (0.999, 1.001)	0.790
Selenium, μg/d	1.002 (0.995, 1.009)	0.587	1.002 (0.995, 1.009)	0.570

^*^Adjusted for age, sex, body mass index, poverty-income ratio, education, ethnicity, smoking, drinking, hypertension, diabetes, and caffeine intake.

**Table 5:** The association between total dietary intakes and PD Risk.

Variable	Unadjusted OR (95% CI)	*p*	Adjusted OR (95% CI)*	*p*
Lycopene, μg/d	1.000 (1.000, 1.000)	0.112	1.000 (1.000, 1.000)	0.110
Vitamin B1, mg/d	1.000 (0.994, 1.007)	0.910	1.001 (0.994, 1.007)	0.870
Vitamin B2, mg/d	1.008 (0.984, 1.033)	0.524	1.009 (0.987, 1.032)	0.427
Niacin, mg/d	1.000 (0.998, 1.002)	0.953	1.000 (0.998, 1.002)	0.920
Vitamin B6, mg/d	1.000 (0.986, 1.014)	0.993	1.000 (0.987, 1.013)	0.979
Folic acid, μg/d	1.000 (1.000, 1.000)	0.424	1.000 (1.000, 1.000)	0.468
Vitamin B12, μg/d	1.000 (0.999, 1.000)	0.373	1.000 (0.999, 1.000)	0.380
Vitamin C, mg/d	0.998 (0.996, 0.999)	0.008	0.998 (0.996, 0.999)	0.008
Vitamin D, μg/d	0.997 (0.987, 1.007)	0.547	0.997 (0.985, 1.008)	0.553
Vitamin K, μg/d	1.000 (1.000, 1.000)	0.452	1.000 (1.000, 1.000)	0.526
Calcium, mg/d	1.000 (0.999, 1.001)	0.896	1.000 (0.999, 1.001)	0.920
Phosphorus, mg/d	1.000 (0.999, 1.002)	0.423	1.000 (0.999, 1.002)	0.507
Magnesium, mg/d	0.999 (0.996, 1.001)	0.240	0.998 (0.996, 1.001)	0.176
Iron, mg/d	1.008 (0.997, 1.018)	0.154	1.008 (0.999, 1.018)	0.088
Zinc, mg/d	0.977 (0.949, 1.006)	0.116	0.979 (0.951, 1.007)	0.141
Copper, mg/d	1.079 (0.966, 1.205)	0.180	1.093 (0.975, 1.224)	0.126
Sodium, mg/d	1.000 (1.000, 1.000)	0.947	1.000 (1.000, 1.000)	0.996
Potassium, mg/d	1.000 (1.000, 1.001)	0.430	1.000 (1.000, 1.001)	0.592
Selenium, μg/d	0.997 (0.990, 1.004)	0.401	0.998 (0.991, 1.005)	0.584

*Adjusted for age, gender, BMI, poverty income ratio, education, race, smoking, drinking, hypertension, diabetes, caffeine intake.

### 3.4 Propensity score matching analysis

99 patients in the PD group were subjected to matching with 396 patients in the no-PD group. After PSM, participant characteristics were effectively balanced between the two groups (except for the type of education, Supplementary Table S2). After PSM, PD patients continued to exhibit potentially higher intake of retinol (*p* = 0.029), vitamin A (*p* = 0.008), and iron (*p* = 0.016) from food. Additionally, the percentage of individuals in the non-PD group using dietary supplements was significantly higher (p = 0.029). Furthermore, individuals with PD consumed significantly less vitamin C (*p* = 0.009) and zinc (*p* = 0.036).

In the logistic regression performed after PSM, it was found that higher dietary iron intake continued to be linked with an elevated risk of PD (OR 1.098, 95% CI 1.031–1.168, *p* = 0.003; Supplementary Table S4). Additionally, an inverse correlation was found between greater zinc intake from food and the risk of PD (OR 0.850, 95% CI 0.757–0.953, *p* = 0.006; Supplementary Table S4). When we examined total dietary intake from food and dietary supplements, we still found a significant negative correlation between higher intake of vitamin C and the risk of PD (OR 0.997, 95% CI 0.995–0.999, *p* = 0.002; Supplementary Table S3). Furthermore, an elevated intake of copper was related to an increased risk of PD (OR 1.527, 95% CI 1.016–2.295, *p* = 0.035). Even after accounting for potential confounding factors, these associations retained their statistical significance.

### 3.5 Subgroup analyses

Supplementary Table S4 presents the results of a subgroup analysis, stratified by gender and age, to examine the association between dietary intakes of vitamin C, vitamin K, iron, zinc, and copper, and the odds of developing PD. A positive correlation between dietary iron intake from food and the risk of PD clearly observed in individuals aged less than 60 years and among males. As for dietary zinc intake, the inverse association was only observed in female.

## 4 Discussion

In this first application of NHANES data to PD risk, we identified dietary intake of zinc, vitamins C and K as potentially protective against PD, while iron and copper intake appears to increase risk.

Our analysis of a large sample of more than 10,000 individuals may help clarify the inconsistent literature on whether vitamin C intake can decrease risk of PD (15–19). For example, a large Swedish cohort study that followed participants for 17 years linked higher dietary intake of vitamins E and C to lower risk of PD (16), but the Singapore Chinese Health Study found no correlation between dietary intake of carotenoids, as well as vitamins A, C, or E, and the risk of PD (17). Our results suggest that deficiency in vitamin C can increase risk of the disease, perhaps because the body cannot buffer oxidative stress (20).

Our work suggesting a negative correlation between dietary intake of vitamin K and PD risk should inspire further work on this topic, since the literature has neglected this potential benefit of vitamin K. We are aware of one study linking deficiency of vitamin K2 with PD progression (21). Vitamin K may reduce risk of PD by scavenging free radicals and thereby dampening injury due to reactive oxygen species that can lead to PD (22, 23).

The observed Inverse relationship between zinc Intake and the PD risk in our present study is in line with the findings from a prior Japanese case–control study including 249 cases and 368 controls (24). Zinc, an antioxidant and anti-inflammatory agent, performs distinctive and sequential functions in counteracting free radicals and safeguarding neurons from oxidative harm (25). An experimental study suggested the advantageous impact of zinc supplementation in a Drosophila PD model (26). In addition, gender may exert an influence on PD risk in the context of lower zinc intake. Nevertheless, additional research is essential to validate the sex-specific effects.

We observed that a higher intake of dietary iron from food sources was associated with an increased risk of PD. This supports the widely held belief that iron dysregulation contributes to PD and parkinsonism (27), and it aligns with the observation that individuals with PD tend to have significantly higher levels of iron in serum than healthy controls (28). Iron is an indispensable trace element within the human body. Nonetheless, its redox activity, along with the generation of free radicals during the interconversion between ferrous (Fe2+) and ferric (Fe3+) iron, also confers toxic properties (29). This redox-active metal generates an excessive amount of reactive oxygen species through the Fenton reaction, leading to pronounced oxidative stress reactions and resultant cellular damage (29). Furthermore, Fe2+ can also interact with the negatively charged C-terminus of α-synuclein, accelerating its aggregation, and the redox reactions of iron can promote the oligomerization of α-synuclein in the substantia nigra (SN) (30, 31). Excessive iron may deposit in the substantia nigra and kill dopaminergic neurons, increasing risk of PD (32). Nevertheless, the literature on the relationship between iron intake and the risk of PD has shown inconsistent findings (33–36), so our relatively large study may help to clarify this question.

Although we found iron intake from food to correlate positively with PD risk, our study did not establish a significant link between total iron intake and the risk of PD. Similarly, a large study including 124,353 participants in the US only identified a positive correlation between the risk of PD and intake of iron from food (36). On the other hand, a prospective study of nearly 390,000 people suggested that higher supplemental iron consumption was linked to greater risk of parkinsonism (37). Furthermore, our subgroup analysis indicates a positive correlation between high iron intake and PD risk in men and individuals aged under 60 years. Notably, two prior studies (38, 39) have similarly identified gender-specific associations, with an increased risk of PD associated with dietary iron intake observed exclusively in men. Our results suggest that both gender and age may be influential factors in the relationship between iron intake and the risk of PD. Menstrual blood loss is a substantial factor that influences iron levels in women (40). Additionally, a prior study (41) observed that the prevalence of anemia increased with age in individuals aged ≥65 years, for both men and women. Future research endeavors are essential to corroborate these associations and to elucidate whether the influence of iron on the risk of PD depends on its form and route of intake.

In our study, we discovered that a higher total intake of copper may be associated with an elevated risk of PD. While a previous meta-analysis (39) did not identify a connection between dietary intake of copper and PD risk, other studies (42–44) have suggested that disruptions in copper homeostasis, which can result in either increased or reduced levels, may contribute to the onset of PD.

It’s important to interpret our findings with caution since the dietary data was obtained from two cross-sectional diet assessments, which are subject to recall bias and do not capture potentially substantial day-to-day variations in dietary consumption. In addition, the study participants were not prospectively diagnosed with PD but simply asked whether they were taking anti-PD medications, which was similar to a previous study (11). This approach may have missed some cases of PD. A third limitation is that we considered nutrients and non-nutrients individually, without accounting for their potential interactions (45). Such interactions may help explain some of the inconsistencies in the literature on dietary risk factors of PD. A fourth limitation is that we collected dietary data on individuals with PD after they had been diagnosed with the disease and were taking medication, which may have confounded our analyses of dietary risk factors. Ultimately, suitably large prospective studies are needed to explore whether and how diet affects risk of PD.

## 5 Conclusion

Our analysis, conducted on a relatively large sample from the United States, indicates that higher iron intake from food and total copper intake are associated with an increased risk of PD. Conversely, increased total dietary intake of vitamins C and vitamins K from food, along with zinc intake, are linked to a reduced risk of PD. These findings should be considered cautiously, and they need to be replicated in other settings, possibly with biomarkers. Further research is necessary to provide a more comprehensive assessment.

## Data availability statement

The original contributions presented in the study are included in the article/Supplementary material, further inquiries can be directed to the corresponding author.

## Ethics statement

The studies involving humans were approved by NCHS Research Ethics Review Board. The studies were conducted in accordance with the local legislation and institutional requirements. Written informed consent for participation was not required from the participants or the participants' legal guardians/next of kin in accordance with the national legislation and institutional requirements.

## Author contributions

LL: Conceptualization, Data curation, Formal analysis, Investigation, Project administration, Software, Validation, Writing – original draft. QS: Project administration, Validation, Writing – review & editing. YB: Data curation, Methodology, Project administration, Software, Writing – review & editing. FX: Investigation, Software, Writing – review & editing. DZ: Project administration, Writing – review & editing. HH: Methodology, Supervision, Writing – review & editing. LT: Supervision, Writing – review & editing. YX: Methodology, Supervision, Writing – review & editing.
