# Supplementary material for: Association between dietary intake and risk of Parkinson’s disease: cross-sectional analysis of survey data from NHANES 2007–2016
Source: Front Nutr. 2023 Dec 15;10:1278128. doi: 10.3389/fnut.2023.1278128 (PMC10773772; doi:10.3389/fnut.2023.1278128)
Supplement: Supplementary file 1 [file Image_1.pdf]

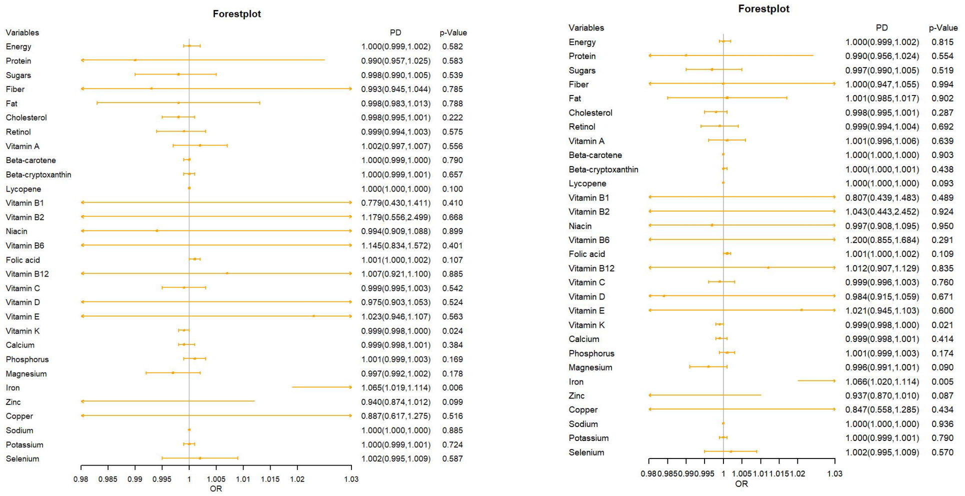

A B

Figure S1. Forest plot of dietary intake from food and risk of Parkinson's disease (PD)

(A =unadjusted, B = adjusted)
